# Identification and validation of cuproptosis‐related molecular clusters in non‐alcoholic fatty liver disease

**DOI:** 10.1111/jcmm.18091

**Published:** 2024-01-03

**Authors:** Changxu Liu, Zhihao Fang, Kai Yang, Yanchao Ji, Xiaoxiao Yu, ZiHao Guo, Zhichao Dong, Tong Zhu, Chang Liu

**Affiliations:** ^1^ Department of General Surgery Fourth Affiliated Hospital of Harbin Medical University Harbin China; ^2^ Beijing Chaoyang Hospital Affiliated to Capital Medical University Beijing China

**Keywords:** cuproptosis, diagnostic, immune, non‐alcoholic fatty liver disease

## Abstract

Non‐alcoholic fatty liver disease (NAFLD) is a major chronic liver disease worldwide. Cuproptosis has recently been reported as a form of cell death that appears to drive the progression of a variety of diseases. This study aimed to explore cuproptosis‐related molecular clusters and construct a prediction model. The gene expression profiles were obtained from the Gene Expression Omnibus (GEO) database. The associations between molecular clusters of cuproptosis‐related genes and immune cell infiltration were investigated using 50 NAFLD samples. Furthermore, cluster‐specific differentially expressed genes were identified by the WGCNA algorithm. External datasets were used to verify and screen feature genes, and nomograms, calibration curves and decision curve analysis (DCA) were performed to verify the performance of the prediction model. Finally, a NAFLD‐diet mouse model was constructed to further verify the predictive analysis, thus providing new insights into the prediction of NAFLD clusters and risks. The role of cuproptosis in the development of non‐alcoholic fatty liver disease and immune cell infiltration was explored. Non‐alcoholic fatty liver disease was divided into two cuproptosis‐related molecular clusters by unsupervised clustering. Three characteristic genes (ENO3, SLC16A1 and LEPR) were selected by machine learning and external data set validation. In addition, the accuracy of the nomogram, calibration curve and decision curve analysis in predicting NAFLD clusters was also verified. Further animal and cell experiments confirmed the difference in their expression in the NAFLD mouse model and Mouse hepatocyte cell line. The present study explored the relationship between non‐alcoholic fatty liver disease and cuproptosis, providing new ideas and targets for individual treatment of the disease.

## 1 INTRODUCTION

Globally, the number of patients with metabolic disorders has been increasing as obesity rates have steadily risen. Non‐alcoholic fatty liver disease (NAFLD) is the hepatic manifestation of metabolic syndrome and is mainly characterized by excessive accumulation of fat in hepatocytes (≥5%) in the absence of excessive alcohol consumption.^1^ Statistics show that around 24% of the world's population is affected by non‐alcoholic fatty liver disease (NAFLD).^2^ The pathological changes of the liver in NAFLD range from simple hepatic steatodegeneration to non‐alcoholic steatohepatitis (NASH), and may even develop into liver fibrosis, cirrhosis and liver cancer in severe cases.^3^

However, the pathogenesis of NAFLD/NASH remains unclear. The classical theory of NASH progression is the ‘two‐hit hypothesis’, but this conventional view is too simplistic to account for all the molecular and metabolic changes in NASH pathogenesis.^4^, ^5^ More recently, oxidative stress has been reported to play a major role in the progression of hepatic steatosis to NASH. Oxidative stress results from an imbalance between the body's antioxidant defences and reactive oxygen species (ROS). ROS are highly reactive molecules and excessive ROS can result in oxidative stress and damage cellular components, such as DNA, proteins and lipid bilayers. The antioxidant defence includes enzymatic antioxidants such as superoxide dismutase (SOD), catalase and glutathione peroxidase (GSH‐PX) and small molecules such as vitamin C and GSH.^6^ However, a highly oxidized environment can aggravate inflammation, fibrosis formation and hepatocyte death, leading to the pathological progression of NAFLD.^7^ The copper ion is essential for a number of biological processes. As a coenzyme factor, Cu^2+^ mainly relies on mitochondrial regulation^8^ to maintain homeostasis. Copper is mainly present in mitochondria in the form of cytochrome c oxidase (COX) and superoxide dismutase (SOD1), which regulate the tricarboxylic acid (TCA) cycle and terminal oxidation. Furthermore, it participates in multiple biological processes such as reduction balance, iron utilization, oxidative phosphorylation and cell proliferation.^9^, ^10^ Programmed cell death (PCD) refers to a genetically regulated cellular suicide, which plays a crucial role in tissue homeostasis and growth and is also involved in several pathological processes.^11^ Various types of programmed cell death have been found to date, including necroptosis and ferroptosis.^11^

Cuproptosis is a newly identified form of programmed cell death that is distinct from other oxidative stress‐regulated death events such as pyroptosis, ferroptosis and necrosis. Mitochondrial stress has been reported to be the main mechanism leading to cuproptosis,^12^ which is characterized by excessive accumulation of mitochondrial fatty acid acylase and depletion of Fe‐S‐cluster proteins. Moreover, a growing number of reports indicate a complex relationship between the regulatory imbalance of copper ions in NAFLD and NASH.^13^ However, the potential regulatory mechanism of cuproptosis in NAFLD remains unclear and requires further exploration. Therefore, NAFLD heterogeneity may be attributed to the molecular characteristics of cuproptosis‐related genes (CRGs).

For the first time, this study systematically investigated the differential expression of CRGs and immune infiltration characteristics between normal subjects and patients with NAFLD. Based on nine differentially expressed CRG profiles, NAFLD patients were divided into two cuproptosis‐related clusters and the immune infiltration differences between the two clusters were investigated. Subsequently, cluster‐specific differentially expressed gene (DEGs) were identified and the enriched biological functions and pathways were elucidated based on cluster‐specific DEGs. In addition, multiple machine learning algorithms and predictive models were constructed to identify patients with different molecular clusters. The performance of the prediction model was verified using nomograms, calibration curves, decision curve analysis (DCA) and external data sets. Finally, a mouse model of NAFLD was established by a high‐fat diet (HFD) to further validate the predictive analysis, providing new insights into the prediction of NAFLD clusters and risk.

## 2 MATERIALS

### 2.1 Data collection and preprocessing

Gene expression profiles were obtained from the Gene Expression Omnibus (GEO) database (http://www.ncbi.nlm.nih.gov/geo) and the NAFLD datasets GSE89632, GSE63067 and GSE48452 were downloaded from the GEO database. All the datasets originated from Homo sapiens. The GSE48452 data set used the GPL11532 data platform, with a total of 73 samples, including 14 normal liver tissue control samples, 27 obese liver tissue samples and 32 NAFLD tissue samples. The GSE89632 dataset used GPL14951 and contained 63 samples, including 24 normal liver control samples and 39 NAFLD tissue samples. All samples were included in the present. In addition, the GSE63067 dataset used GPL14877 and contained 18 samples, including 7 normal liver control samples and 11 NAFLD tissue samples. In this study, dataset GSE48452 was used as the validation set. The GSE63067 and GSE89632 data sets were merged and the batch effect was removed by the SVA package.^14^ Finally, the R package ‘Limma’ was used for analysis.^15^ The flow chart of this study is displayed in Figure S1.

### 2.2 Evaluating the immune cell infiltration

Cibersort was used with the LM22 genetic characteristic matrix (https:/cibersort.stanford.edu/) algorithm, based on the gene expression profile assessment subtype of the immune system cells within each sample. Furthermore, the *p*‐value of the backfold product of each sample was calculated based on Monte Carlo sampling, and the Wilcoxon rank sum test was used to estimate differences in immune cell abundance between groups. In this study, *p* < 0.05 was considered statistically significant.

### 2.3 Correlation analysis of CRGs and immune cell infiltration

To further confirm the relationship between CRG and NAFLD‐associated immune cell properties, the relationship between CRG expression and the relative proportion of immune cells was examined. Spearman's correlation coefficient and its associated *p*‐value were used to evaluate the correlation, with a *p*‐value<0.05 indicating a significant association. Finally, the results were displayed using the ‘corrplot’ R package (version 0.92).

### 2.4 Unsupervised clustering of NAFLD patients

Cuproptosis‐related genes were derived from previous studies.^16^ Based on the expression profiles of 9 cuproptosis genes with significantly different expressions, an unsupervised cluster analysis^17^ was performed to classify 50 NAFLD samples into different clusters through 1000 iterations of the k‐means algorithm. A maximum subtype number k (*k* = 9) was selected, and the optimal subtype number was comprehensively evaluated based on the cumulative distribution function (CDF) curve, consensus matrix and consensus cluster score (>0.9). PCA (Principal Component Analysis) analysis showed differences in the distribution of cuproptosis among subtypes and was visualized using the ‘ggplot2’ software package.

### 2.5 Gene set variation analysis (GSVA) analysis

The enrichment analysis of different CRG clusters was analysed by the GSVA (Version 2.11) R package. Then, the files ‘c2.cp.kegg.v7.4.symbals’ and ‘c5.go.bp.v7.5.1.symbols’ were obtained from the MSigDB database for further GSVA analysis. The ‘LIMMA’ R package (version 3.52.1) was used to determine differential expression pathways and biological functions by comparing GSVA scores between different CRG clusters.

### 2.6 Weighted gene co‐expression network analysis (WGCNA)

The R package^17^ ‘WGCNA’ (version 1.70.3) was used to identify co‐expression modules. To ensure the accuracy of the study, we first grouped the samples and eliminated outliers. A soft threshold from 1 to 20 was used for topology calculation to determine the optimal soft threshold. When the minimum module size was set to 100, a ‘dynamic tree cutting’ algorithm was used to group genes with similar patterns into modules. Finally, Pearson correlation analysis was performed to calculate the correlation between modules and traits. Based on the correlation between modules and clinical features, the most relevant module to the disease is selected as the key module.

### 2.7 Construction of multiple machine learning prediction models

Based on two different CRG clusters, the ‘caret’ R package (version 6.0.91) was applied to build machine learning models, including the random forest model (RF), support vector machine model (SVM), generalized linear model (GLM) and eXtreme Gradient Boosting (XGB). RF is a machine learning technique that employs multiple independent decision trees for classification or regression predictions.^18^ In contrast, the support vector machine algorithm generates a hyperplane with a maximum margin in the feature space to distinguish positive from negative instances.^19^ The generalized linear regression model is an extension of the multiple linear regression model that flexibly estimates the relationship between normally distributed correlated features and categorical or continuous independent features.^20^ Furthermore, XGB is a gradient boosting‐based boost tree ensemble that enables careful comparison between classification error and model complexity.^21^ Different clusters were used as the response variable, while cluster‐specific DEGs were selected as explanatory variables. The 50 NAFLD samples were randomly divided into the training set (70%, *N* = 35) and the validation set (30%, *N* = 15). The parameters of these models were automatically tuned using web searches, and all of these machine learning models were run with preset parameters and evaluated by 5‐fold cross‐validation. The ‘Dalex’ package (version 2.4.0) was developed to interpret the above four machine learning models and to visualize the distribution of residuals and the importance of features in these machine learning models. The area under the ROC curve was displayed using the ‘proc’ R package (version 1.18.0). Therefore, the most suitable machine learning model was evaluated and the five most important variables were identified as the main predictor genes associated with NAFLD.

### 2.8 Analysis of the diagnostic value of biomarkers

The receiver operating characteristic (ROC) curve was generated using the R package ‘pROC’ and the area under the ROC curve (AUC) value was calculated. The ability of the key predictor genes to distinguish NAFLD from non‐NAFLD was externally validated using the GSE48452 dataset.

### 2.9 Construction and validation of nomogram models

Eigengenes were combined to construct nomograms using the ‘rms’ R package. Subsequently, the accuracy of the nomogram was assessed using a calibration curve, and the clinical application value of the chart was evaluated by decision curve analysis.

### 2.10 Establishment of NAFLD animal model

Six‐week‐old male C57BL/6J mice (*n* = 14) weighing about 22 g were provided by Liaoning Changsheng Biotechnology Co., Ltd., and were fed with a standard diet and standard 12 h:12 h light/dark cycle until 8 weeks old. Then, the mice were randomly divided into two groups: one group was fed a high‐fat diet (research diet D12451, 45 kcal saturated fat) to induce NAFLD (*n* = 7), while the other group was fed a normal diet (5% fat, 53% carbohydrate, 23% protein), serving as the control group (*n* = 7). The mice were fed for 16 weeks, and one mouse was randomly selected from each group and was sacrificed by cervical dislocation. Liver samples were collected, fixed in 4% formalin for 24 h and dehydrated with a series of ethanol solutions. Liver tissues were then embedded in paraffin and sectioned at a thickness of 4 μM. The sections were stained with haematoxylin and eosin (H&E). All human and animal studies were approved by the Medical Ethics Committee of the Fourth Affiliated Hospital of Harbin Medical University, and all methods were performed in accordance with relevant guidelines and regulations.

### 2.11 Cell culture and treatment

Mouse hepatocyte cell line (AML12) were purchased from Procell Life Science and Technology (Wuhan, China), maintained and propagated in DMEM/F‐12 with 10% FBS, ITS Liquid Media Supplement (Sigma) and 0.1 μM dexamethasone at 37°C incubators containing 5% CO_2_. To establish the in vitro NAFLD cell model, AML12 were cultured in the presence or absence of 1 mM free fatty acids (FFA, containing oleic acid and palmitic acid at a 2:1 volume ratio) for 48 h and then used for the indicated assays.

### 2.12 Oil red O staining

Oil Red O staining was applied to assess lipid droplet formation in AML12 cells according to a previously described method.^22^ Briefly, cells were washed twice with PBS, fixed in 4% paraformaldehyde for 0.5 h and stained for 30 min with a 0.5% Oil Red O solution in 60% isopropanol. The cells were washed with PBS before analysis. The images were captured under an inverted microscope (Olympus Corporation) at 100× magnification.

### 2.13 Determination of hepatic triglycerides and total cholesterol

Hepatic triglyceride (TG) and liver cholesterol (TC) were measured by Beijing Solarbio ScienceTechnology Co., Ltd. The experimental procedure is briefly described below. Approximately 50 mg of the liver sample was excised, homogenized and then lysed in lysis buffer for 10 min. After centrifugation at 2000 ***g*** for 5 min, the supernatant was divided into two parts: one part was used to determine liver triglyceride (TG) and liver cholesterol (TC), and the other part was used to evaluate protein concentration. Protein quantification was performed using a BCA protein assay kit (P0011, Beyotime, Shanghai, China). Finally, hepatic triglycerides (TG) and hepatic cholesterol (TC) were normalized by protein concentration.

### 2.14 Quantitative RT‐PCR analysis

Total RNA was extracted from liver tissue and AML12 cells using Trizol reagent (Invitrogen) and reverse transcribed using the PrimeScript RT kit (Takara, Tokyo, Japan) according to the manufacturer's instructions. Real‐time quantitative polymerase chain reaction was performed on QuantStudio 3 (Thermo Fisher Scientific China) using SYBR Green PreMix Ex Taq (Takara, Japan), and data analysis was performed using the $2^{-\Delta \Delta C_{t}}$ method, with β‐Actin as the internal control for normalization. The primer sequences used in RT‐PCR are displayed in Table 1.

**TABLE 1:** Primers used for RT‐PCR analysis.

Gene symbol	Species	Forward primer	Reverse primer
β‐Actin	Mouse	GTGACGTTGACATCCGTAAAGA	GCCGGACTCATCGTACTCC
ENO3	Mouse	GAACTCCGAGATGGAGACAAAG	GGACCTAGAGTCTTGTTGATGTG
SLC16A1	Mouse	TACCGGGTGTTCATTGGTGG	TTTGCCAACCACTCCCTACC
LEPR	Mouse	ACCGAGGAATCGTTCTGCAA	GCAGCTATCACATAAAGAAATTCCC

### 2.15 Statistical analysis

All data are presented as mean ± standard deviation. Statistical analysis between groups was performed using an unpaired two‐tailed Student's *t*‐test. In this study, *p* < 0.05 was considered statistically significant. All analyses were performed using GraphPad Prism 8 (GraphPad Software, San Diego, USA).

## 3 RESULTS

### 3.1 Cuproptosis‐associated gene dysregulation and immune cell infiltration in patients with non‐alcoholic fatty liver disease

**FIGURE 1:**
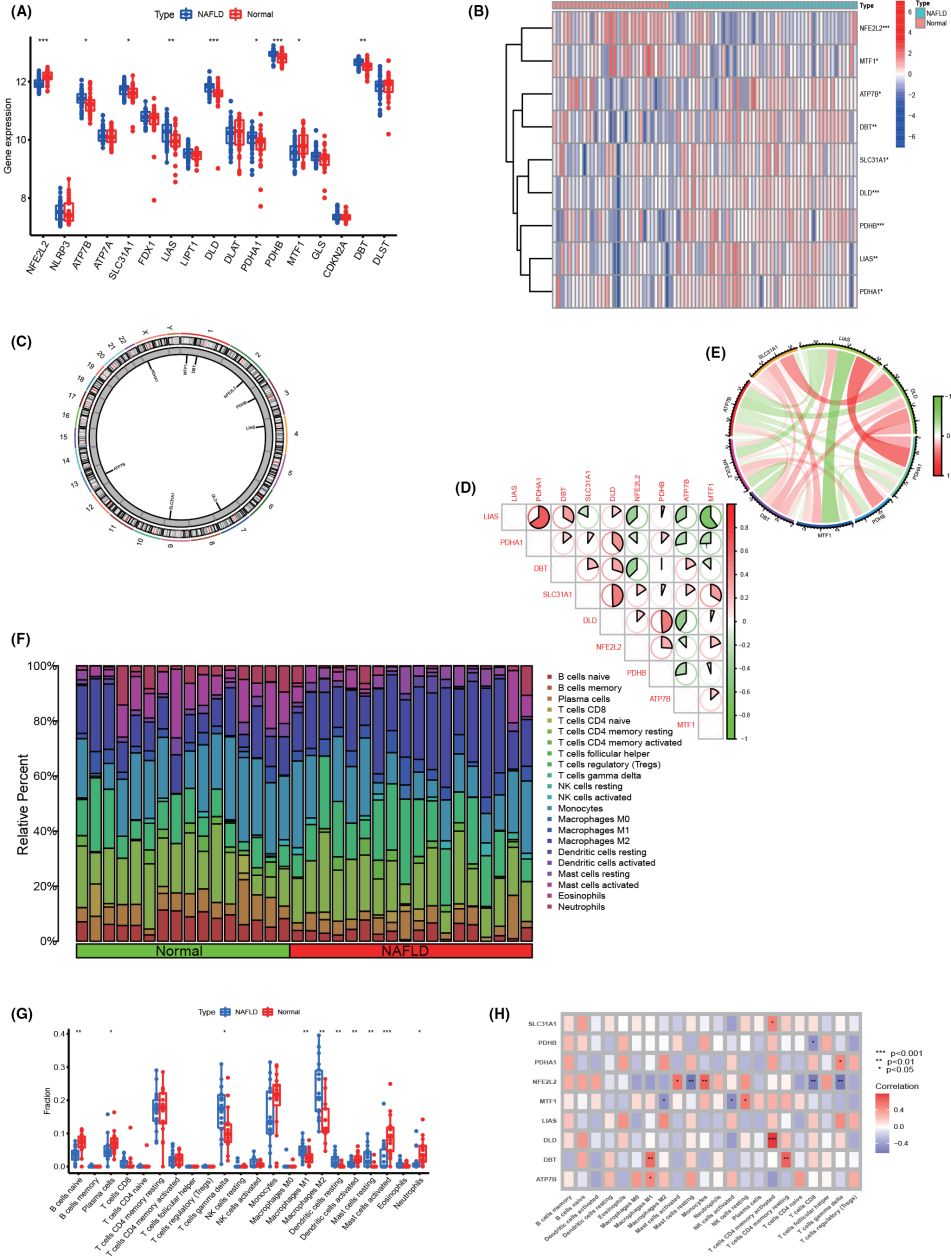
CRGs expression levels in NAFLD. (A) Boxplots showed the expression of 17 CRGs between NAFLD and non‐NAFLD controls. *p < 0.05, **p<0.01,***p < 0.001, ns, no significance. (B) The expression patterns of 9 CRGs were presented in the heatmap. (C) The location of 9 CRGs on chromosomes. (D) Correlation analysis of 9 differentially expressed CRGs. (E) Gene relationship network diagram of 9 differentially expressed CRGs. (F) The relative abundances of 22 infiltrated immune cells between NAFLD and non‐NAFLD controls. (G) Boxplots showed the differences in immune infiltrating between NAFLD and non‐NAFLD controls. *p < 0.05, **p < 0.01***p < 0.001, ns, no significance. (H) Correlation analysis between 9 differentially expressed CRGs and infiltrated immune cells.

To elucidate the biological functions of cuproptosis regulators in the development and progression of non‐alcoholic fatty liver disease (NAFLD), the expression profiles of 19 cuproptosis‐associated genes between NAFLD and non‐NAFLD controls were first assessed using a merged dataset of GSE89632 and GSE63067. The dataset consisted of 50 NAFLD tissues and 31 normal liver tissues. Figure S2 shows the data before batch correction (A and B) and after batch correction (C and D), suggesting the successful elimination of the batch effect from the pooled data. A total of nine genes were selected as cuproptosis regulators with more significant expression differences. Some showed elevated expression in NAFLD patients compared to non‐NAFLD controls, including ATP7B, SLC31A1, LIAS, DLD, PDHA1, PDHB and DBT. In contrast, NFE2L2 and MTF1 showed decreased expression in NAFLD tissues compared to non‐NAFLD controls (Figure 1A–C). Moreover, correlation analysis was performed between the differentially expressed cuproptosis genes, demonstrating a strong synergistic effect between LIAS and PDHA1, and a clear antagonism between MTF1 and LIAS. In addition, the correlation between these cuproptosis genes was further investigated, as shown in Figure 1D,E. Immune infiltration analysis was performed to visualize differences in the proportions of 22 infiltrating immune cell types between NAFLD controls and non‐NAFLD control subjects using the CiberSort algorithm (Figure 1F). The results showed higher levels of γ‐delta T cells, M1 macrophages, M2 macrophages, resting dendritic cells and resting mast cells in NAFLD samples compared to the control group (Figure 1G), suggesting that alterations in the immune system may be involved in the development of NAFLD. Furthermore, correlation analysis results revealed that activated CD4 memory T cells, M2 macrophages and activated mast cells were all associated with cuproptosis‐related genes (Figure 1H). These results suggest that CRGs may be a key factor regulating the occurrence and immune infiltration status of NAFLD patients.

### 3.2 Identification of cuproptosis clusters in NAFLD

**FIGURE 2:**
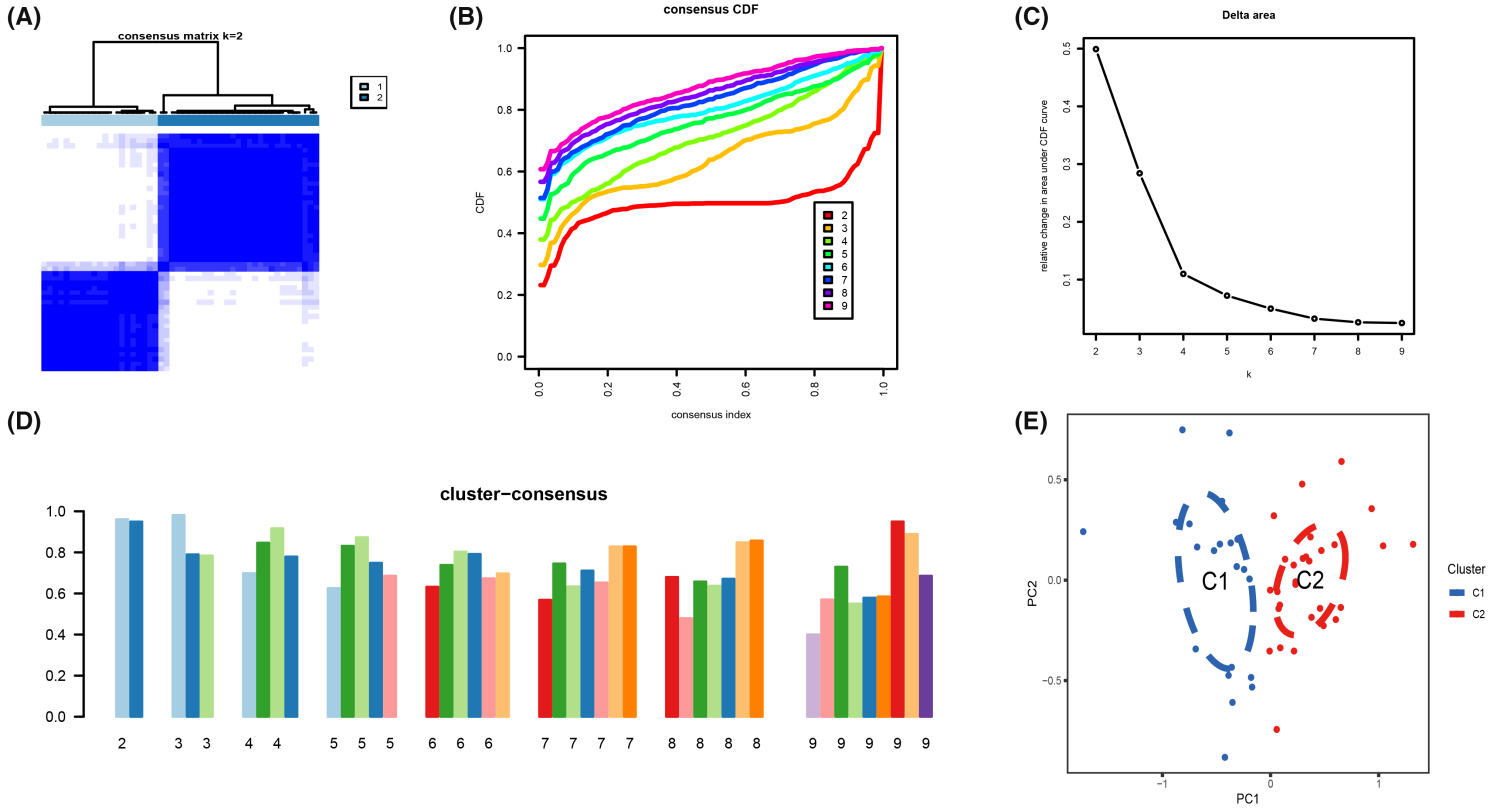
Identification of cuproptosis‐related molecular patterns in NAFLD. (A) Consensus clustering matrix when *k* = 2. (B–E) Representative CDF curves when *k* = 2 to 9. (C) Relative alterations in CDF delta area curves. (D) Consensus score in each subtype when *k* = 2 to 9. (E) PCA diagram demonstrates that NAFLD patients are classified into two distinct subtypes.

Additionally, 50 NAFLD samples were grouped based on the expression profiles of 9 differentially expressed CRGs using a consensus clustering algorithm to identify the expression patterns of genes associated with copper death in NAFLD. The number of clusters was most stable when the *k* value was set to 2 (*k* = 2) (Figure 2A), and the CDF curve fluctuated within the smallest range of consensus index 0.2 to 0.6 (Figure 2B). When *k* = 2 ~ 9, the area under the CDF curve showed a difference between the two CDF curves (*k* and *k*‐1) (Figure 2C). Furthermore, when *k* = 2, the concordance scores for each subtype >0.9. (Figure 2D). Therefore, the 50 NAFLD patients were divided into two groups, including group 1 (*n* = 21) and group 2 (*n* = 29). The results of the PCA (Principal Component Analysis) analysis showed significant differences between the two clusters (Figure 2E).

### 3.3 Differential expression of genes regulated by cuproptosis and immune infiltration signatures of associated cuproptosis clusters

**FIGURE 3:**
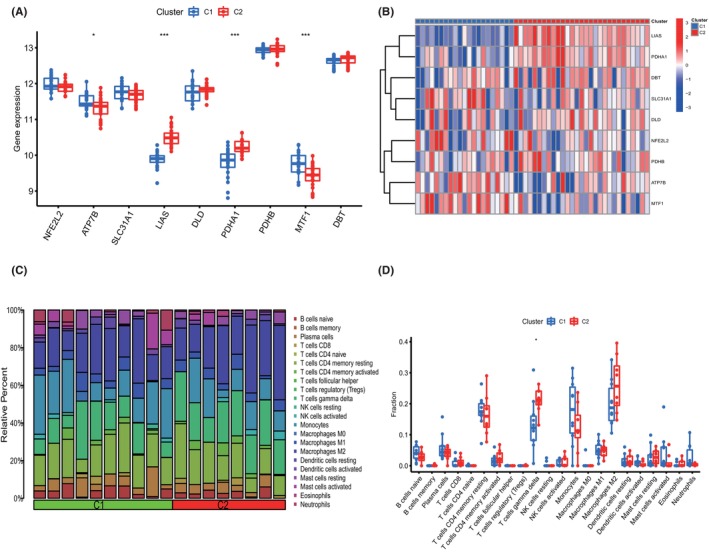
Identification of the differentiation of feature genes and immune characteristics between two cuproptosis clusters. (A) Boxplots show the expression of 9 characteristic genes between cuproptosis subtypes. *p < 0.05, ***p < 0.001, ns, no significance. (B) Heatmap reveals the differential expression of 9 characteristic genes between cuproptosis subtypes. (C) The relative abundances of 22 infiltrated immune cells between two cuproptosis clusters. (D) Boxplots show the differences in infiltrated immune cells between cuproptosis subtypes. *p < 0.05, ns, no significance.

The expression differences of 9 CRGs between Cluster 1 and Cluster 2 were comprehensively assessed to further explore the molecular features between subgroups. Cuproptosis Cluster1 showed high expression of MTF1 and ATP7B, while cuproptosis Cluster2 was characterized by enhanced expression of LIAS and PDHA1 (Figure 3A,B). In addition, the results of the immune infiltration assay revealed differences in the immune microenvironment between cuproptosis Cluster1 and Cluster2 (Figure 3C). Among them, the most significant changes were γ‐Delta T cells, which had a higher proportion in Cluster2 (Figure 3D).

### 3.4 Construction of gene weighted co‐expression module and gene screening

**FIGURE 4:**
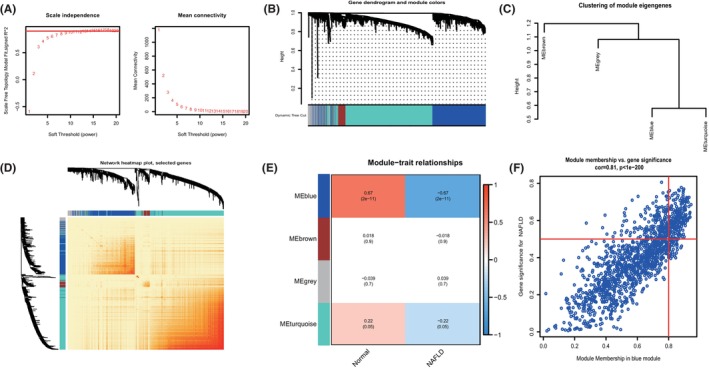
Co‐expression network of differentially expressed genes in NAFLD. (A) The selection of soft threshold power. (B) Cluster tree dendrogram of co‐expression modules. Different colours represent distinct co‐expression modules. (C) Representative of clustering of module eigengenes. (D) Representative heatmap of the correlations among 4 modules. (E) Correlation analysis between module eigengenes and clinical status. Each row represents a module; each column represents a clinical status. (F) Scatter plot between module membership in the blue module and the gene significance for NAFLD.

**FIGURE 5:**
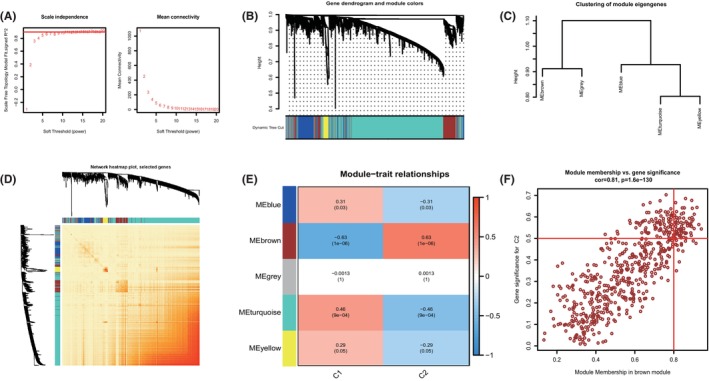
Co‐expression network of differentially expressed genes between the two cuproptosis clusters. (A) The selection of soft threshold power. (B) Cluster tree dendrogram of co‐expression modules. Different colours represent distinct co‐expression modules. (C) Representative of clustering of module eigengenes. (D) Representative heatmap of the correlations among 5 modules. (E) Correlation analysis between module eigengenes and clinical status. Each row represents a module; each column represents a clinical status. (F) Scatter plot between module membership in the brown module and the gene significance for Cluster 2.

The WGCNA algorithm was used to establish co‐expression networks and modules in normal controls and individuals with NAFLD to identify the key gene modules associated with NAFLD. Co‐expressed gene modules were identified with the soft power value set to 9 and the scale‐free R2 set to 0.9 (Figure 4A **)**. The dynamic cut algorithm was used to obtain 4 co‐expressed gene modules with different colours, and a topological overlap matrix (TOM) heat map was produced (Figure 4B–E). Subsequently, these genes in the 4 colour modules were applied sequentially to analyse the similarity and contiguity of the module‐clinical signature (control and NAFLD) co‐expression. The results revealed that the blue module was most closely related to NAFLD and included 1330 genes (Figure 4F). In addition, a strong positive correlation was found between the blue modules and module‐associated genes. In addition, the WGCNA algorithm was used to analyse key gene modules closely related to cuproptosis genes. *β* = 9 and R2 = 0.9 were screened as the most suitable soft threshold parameters for building scale‐free networks (Figure 5A). Five modules were identified as significant modules, and heatmaps were generated to depict the TOMs of all module‐associated genes (Figure 5B–E). Module‐clinical feature (Cluster1 and Cluster2) relationship analysis revealed a high correlation between brown modules (556 genes) and NAFLD clusters (Figure 5F). Moreover, correlation analysis showed that brown module genes were significantly correlated with selected modules (Figure 5F).

### 3.5 Identification of cluster‐specific DEGs and functional annotation

**FIGURE 6:**
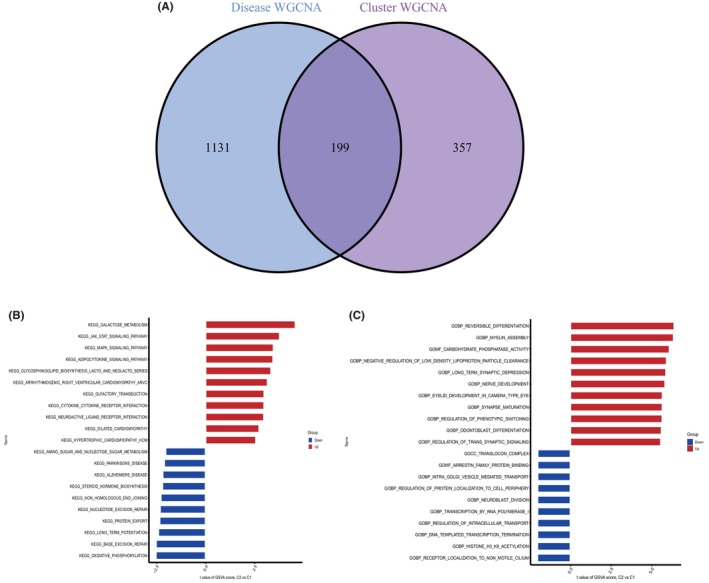
Identifying cluster‐specific DEGs and biological characteristics between two cuproptosis clusters. (A) Intersection of module‐associated genes of a subset of cuproptosis genes with those of NAFLD and non‐NAFLD individuals. (B) Differences in hallmark pathway activities between Cluster1 and Cluster2 samples ranked by *t*‐value of GSVA method. (C) Differences in biological functions between Cluster1 and Cluster2 samples ranked by *t*‐value of GSVA method.

A total of 199 cluster‐specific DEGs were identified by analysing the intersection of module‐associated genes of a subset of cuproptosis genes with those of NAFLD and non‐NAFLD individuals (Figure 6A). GSVA analysis was used to further explore the functional differences between the two clusters associated with cluster‐specific DEGs. The results showed that the galactose metabolism pathway, JAK–STAT signalling pathway, MAKP signalling pathway and adipocytokines signalling pathway were upregulated in Cluster2, while oxidative phosphorylation, base excision repair, steroid hormone biosynthesis pathway and amino sugar and nucleotide sugar metabolism were upregulated in Cluster1 (Figure 6B). Moreover, the functional enrichment results indicated that Cluster2 was associated with the regulation of transsynaptic signalling, maturation of synapses and carbohydrate phosphatase activity (Figure 6C).

### 3.6 Machine learning model construction and evaluation

**FIGURE 7:**
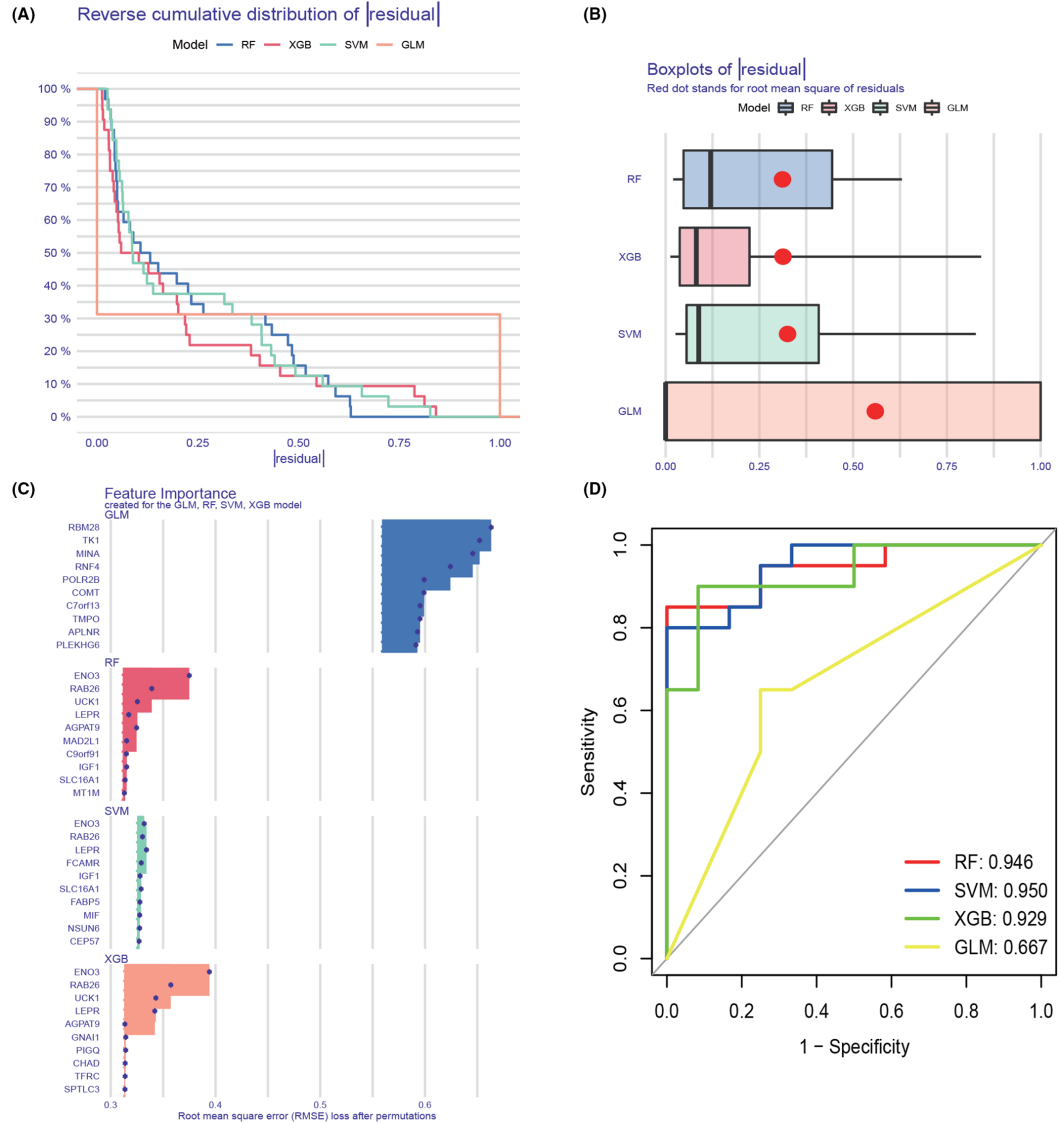
Construction and evaluation of RF, SVM, GLM and XGB machine models. (A) Cumulative residual distribution of each machine learning model. (B) Boxplots showed the residuals of each machine learning model. The red dot represents the root mean square of residuals (RMSE). (C) The important features in RF, SVM, GLM and XGB machine models. (D) ROC analysis of four machine learning models based on 5‐fold cross‐validation in the testing cohort.

**FIGURE 8:**
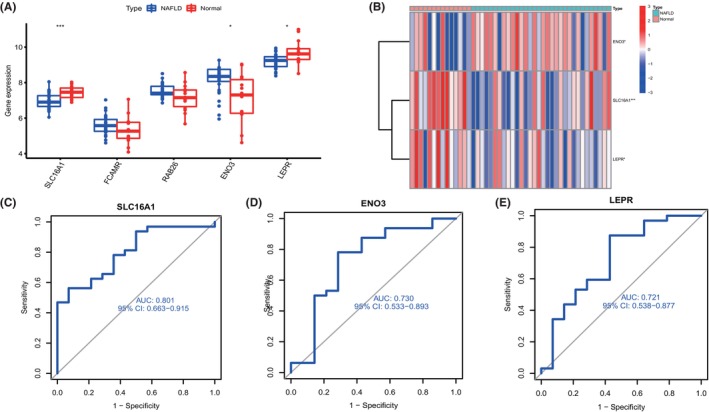
Validation of correlation analysis based on the GSE48452 dataset. (A) Dataset GSE48452 was used to validate the expression of SLC16A1, FCAMR, RAB26, ENO3 and LEPR, the results of which were presented as box plots. *p < 0.05, ***p < 0.001, ns, no significance. (B) The expression of SLC16A1, ENO3 and LEPR were presented in the heatmap. (C–E) The diagnostic effectiveness of the biomarkers for NAFLD by ROC analysis.

**FIGURE 9:**
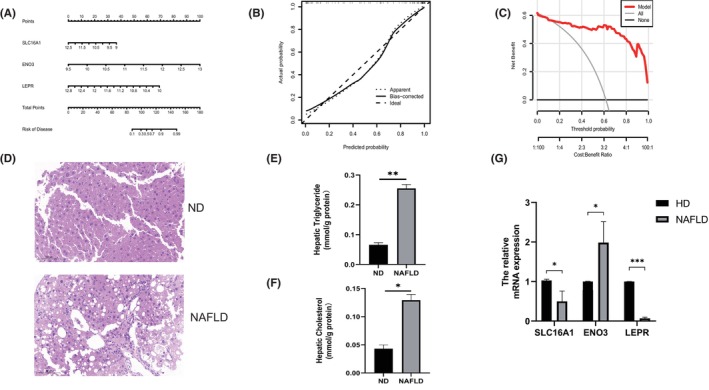
Validation of the model and the expression of SLC16A1, ENO3 and LEPR in HFD diet mice (A) Construction of a nomogram for predicting the risk of NAFLD clusters based on the SLC16A1, ENO3 and LEPR. (B, C) Construction of calibration curve (B) and DCA (C) for assessing the predictive efficiency of the nomogram model. (D) Haematoxylin and eosin (H & E) staining of liver slices. (E) Hepatic triglyceride (TG) levels. (F) Hepatic cholesterol (TC) levels. (G) Genes mRNA expression of SLC16A1, ENO3 and LEPR in hepatic Values are shown as the mean ± s.d. *p < 0.05,**p<0.01,***p<0.001 versus ND.

In total, 199 genes of module‐related genes from individuals with and without NAFLD were intersected based on the subset of cuproptosis genes to further identify cluster‐specific genes with high diagnostic value. Subsequently, four well‐established machine learning models (random forest model, support vector machine model, generalized linear model and extreme gradient boosting) were constructed. The four machine learning models were interpreted by the R package ‘Dalex’ and the distribution of the residuals of each model in the test set was plotted. The RF and SVM machine learning models yielded relatively low residuals (Figure 7A,B). Then, the top 10 significant feature variables of each model were ranked by root mean square error (RMSE) (Figure 7C). Furthermore, the discriminative performance of the four machine learning algorithms was tested and evaluated on the test set by computing receiver operating characteristic (ROC) curves based on fivefold cross‐validation, and the machine learning model with the largest area under the curve was selected. The support vector machine model (SVM) machine learning model showed the largest area under the ROC curve (AUC = 0.950, Figure 7D), demonstrating that this algorithm had the best performance in discriminating patients from different clusters. According to these results, the five most significant variables (SLC16A1, FCAMR, RAB26, ENO3 and LEPR) were selected from the SVM model as predictive genes for further analysis. The expression differences of the signature genes were then validated in dataset GSE48452, which was derived from 32 NAFLD patients and 14 normal population controls. The results revealed that the expression differences of SLC16A1, ENO3 and LEPR were more obvious in the validation set, with SLC16A1 and LEPR being decreased in NAFLD, and ENO3 increased in NAFLD (Figure 8A,B). ROC analysis was further performed, and the results showed its good efficiency in the diagnosis of NAFLD. SLC16A1 (Figure 8C, AUC = 0.801), LEPR (Figure 8E, AUC = 0.721), ENO3 (Figure 8D, AUC = 0.730). A nomogram was built to predict NAFLD progression to further evaluate the predictive power of the SVM model. In the nomogram, the formula for each characteristic variable corresponds to a point value, and the total value corresponds to multiple NAFLD risks, obtained by summing the values of all characteristic variables (Figure 9A). The standard curve confirmed the ability of the nomogram to accurately assess the progression of NAFLD (Figure 9B). Additionally, decision curve analysis indicated that the nomogram may provide clinical benefit to patients with NAFLD (Figure 9C). Collectively, these results suggest that this subset of cuproptosis‐associated genes can accurately distinguish NAFLD from non‐NAFLD cases.

### 3.7 Verification of the expression level of Hub genes in vivo and vitro

**FIGURE 10:**
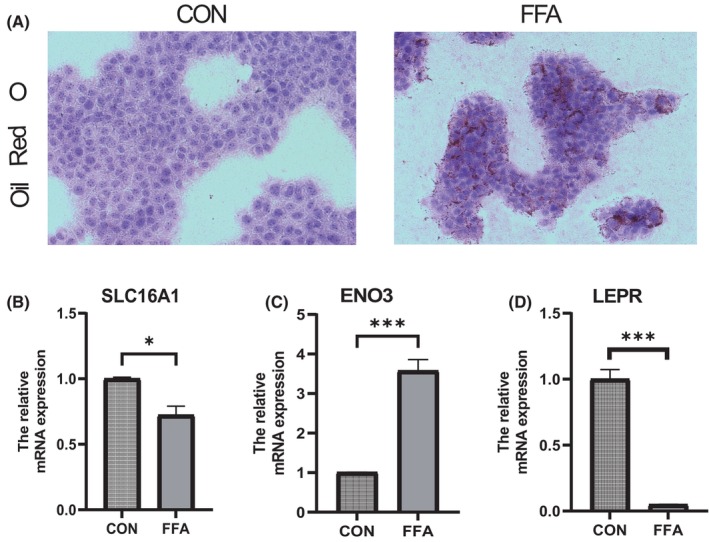
Model gene mRNA expression levels were verified in the AML12 cells. β‐Actin was controlled. (A) Oil red O staining of cells. Magnification ×100. (B–D) Relative mRNA levels of (B) SLC16A1, (C) ENO3 and (D) LEPR. Relative mRNA levels were normalized to those of β‐Actin. Values are shown as the mean ± s.d. *p < 0.05; **p < 0.01, ***p < 0.001.

In order to further verify the expression of genes ENO3, SLC16A1 and LEPR, a NAFLD mouse model was constructed. Therefore, the mice were divided into two groups: the ND group (normal diet) and the NAFLD group (16‐week HFD diet). H&E staining showed that HFD could induce the accumulation of lipid droplets in mouse liver cells, showing no obvious inflammatory reaction (Figure 9D). Furthermore, liver triglyceride levels and cholesterol levels were significantly elevated in mice fed a high‐fat diet for 16 weeks (Figure 9E,F). Compared with the normal diet group, decreased expression of SLC16A1 and LEPR was observed in mice fed HFD, as well as increased expression of ENO3 (Figure 9G). Meanwhile, the in vitro studies were performed. Alpha mouse liver 12 (AML12) cells were treated with OA (oleate) and PA (palmitate) to induce fat accumulation, which indicates the features of hepatic steatosis. After FFA induction, AML12 cells sent a higher level of fat accumulation (Figure 10A), and the expression of ENO3 increased (Figure 10C), while the expression of SLC16A1 and LEPR decreased (Figure 10B,D).

## 4 DISCUSSION

Non‐alcoholic liver disease (NAFLD) is the most common chronic liver disease. Nevertheless, the mechanisms of steatosis in NAFLD remain incompletely understood. Recent research has shown that minerals such as iron, copper, zinc and selenium play important roles in various biochemical processes. In addition, studies on ferroptosis and cuproptosis have further emphasized the importance of intracellular mineral homeostasis. However, mineral imbalances are common in patients with NAFLD and related diseases, and hepatic copper deficiency in NAFLD patients can lead to more pronounced steatosis, NASH and metabolic symptoms.^23^ A large cohort case–control study investigated the relationship between the severity of NAFLD and low blood copper concentrations in men.^24^ As copper is involved in mitochondrial function and fatty acid peroxisome β‐based oxidation, copper deficiency can lead to mitochondrial dysfunction and oxidative stress.^25^, ^26^ The mitochondrial morphology of copper‐deficient rat hepatocytes was abnormally enlarged.^26^ Furthermore, direct experiments also showed that rats fed with a copper‐deficient diet developed spontaneous liver steatosis.^27^ Interestingly, these studies aimed to determine the relationship between mineral disturbances and pathological features of NAFLD, including oxidative stress, mitochondrial dysfunction, inflammatory response and fibrogenesis.^12^ Cuproptosis is a recently reported copper‐dependent cell death. However, the pathogenesis and regulation of cuproptosis in various diseases have not been investigated in detail. Therefore, this study aimed to elucidate the specific roles of cuproptosis‐associated genes in the NAFLD phenotype and immune system infiltration. In addition, the clusters of NAFLD were predicted by using cuproptosis‐related genes for demarking.

This study found 19 genes associated with cuproptosis (CRGs), including NFE2L2, NLRP3, ATP7B, ATP7A, SLC31A1, FDX1, LIAS, LIPT1, LIPT2, DLD, DLAT, PDHA1, PDHB, MTF1, GLS, CDKN2A, DBT, GCSH and DLST. First, the expression profile of cuproptosis‐related genes in the liver of normal people and NAFLD patients was explored. Some cuproptosis genes were significantly differentially expressed in NAFLD patients compared with normal people (NFE2L2, ATP7B, SLC31A1, LIAS, DLD, PDHA1, PDHB, MTF1, DBT), suggesting that these cuproptosis‐related genes play an important role in the development of NAFLD. The correlation analysis results of these genes were performed, strong synergistic effect between LIAS and PDHA1, while MTF1 and LIAS showed significant antagonism. The results of the immune infiltration analysis revealed increases in γ‐ The levels of Delta T cells, M1 macrophages, M2 macrophages, resting dendritic cells and resting mast cells in the liver tissue of NAFLD patients, suggesting that changes in the immune system may be the main cause of NAFLD. Previous studies have also shown that macrophages participate in the inflammatory response of NASH and have a relatively unique phenotype and function.^28^, ^29^ M1 macrophages present antigens and secrete pro‐inflammatory cytokines, while M2 macrophages secrete inhibitory cytokines IL‐10 and transforming growth factor‐β (TGF‐β), and mannose receptor (Mrc) to down‐regulate the immune response.^30^, ^31^ Chronic inflammation, cancer and NAFLD are mostly affected by abnormal regulation of M1/M2 macrophages.^32^ The correlation analysis results demonstrated that activated CD4 memory T cells, M2 macrophages and activated mast cells were all associated with cuproptosis‐related genes (Figure 2H). These results suggest that CRGs may be key factors regulating disease development and immune infiltration status in NAFLD patients. In addition, a consensus clustering algorithm was applied to group 39 NAFLD samples according to the expression profiles of 9 significantly differentially expressed CRGs to illustrate the distinct regulatory patterns of cuproptosis gene clusters in NAFLD patients. In this process, two different cuproptosis‐related clusters were determined. cuproptosis Cluster2 was characterized by the enhanced expression of LIAS and PDHA1, and the two clusters also affected the immune microenvironment. γ‐ Delta T cells accounted for a high proportion in Cluster2. According to previous studies, γδ T17 cells aggravate the progress of NAFLD.^33^, ^34^ Furthermore, Cluster2 was mostly enriched in the MAKP signalling pathway, the JAK–STAT signalling pathway and the galactose metabolism pathway. These two pathways have also been shown by previous researchers to be related to the activation and inflammation of the immune system,^35^, ^36^ and may play a role in the progress of NAFLD.

Machine learning methods^37^, ^38^ were applied to investigate novel disease diagnostic markers, and the predictive performance of the four machine learning classifiers (RF, SVM, GLM and XGB) was compared according to the expression profiles of cluster‐specific DEGs. The results revealed that the SVM‐based predictive model had the highest predictive effect in the test cohort (AUC = 0.950), indicating that SVM‐based machine learning could effectively predict NAFLD clusters. Subsequently, five important variables (SLC16A1, FCAMR, RAB26, ENO3 and LEPR) were selected to construct a 5‐gene‐based SVM model. Three key genes (SLC16A1, ENO3 and LEPR) were identified by further validation and screening using an external data set. SLC16A1 is a well‐studied member of the SLC16A family. Research has shown that SLC16A1 is distributed in almost all human tissues and is overexpressed in many cancers. In addition, up‐regulation of SLC16A1 is associated with poorer prognosis in various cancer types^39^, ^40^; membrane monocarboxylate transporter 1 (Membrane Monocarboxylate Transporter 1, SLC16A1/MCT1) plays an important role in hepatocyte homeostasis and drug action and is involved in liver pathology.^41^ Enolase 3 (ENO3) encodes enolase β subunits, which are distributed in various tissues, including the liver, lung, bone and heart, and has been proven to accelerate the accumulation of hepatic cholesterol ester caused by cholesterol ester synthesis.^42^ Moreover, the up‐regulation of ENO3 in the liver may promote the progress of NASH by increasing GPX4 expression and the negative regulation of ferroptosis by lipid accumulation.^43^ Leptin receptor (LEPR) is a single transmembrane domain receptor in the cytokine receptor family. Leptin controls satiety and maintains the balance of body energy by binding with it.^44^ Relevant studies have reported that the obesity gene LEPR is related to NAFLD.^45^, ^46^ In NAFLD patients, LEPR polymorphism was found to be associated with obesity parameters, insulin resistance and blood glucose levels.^47^ Furthermore, the polymorphism of rs1137100 and rs1137101 in the LEPR locus has been related to an increased risk of NAFLD and NASH. Rs1137100 has also been proven to be related to simple steatosis and NASH.^48^

Finally, SLC16A1, ENO3 and LEPR were used to build a nomogram model to diagnose the NAFLD cluster. The model was found to have significant predictive power, suggesting its clinical utility. In addition, a NAFLD disease model was constructed with C57BL/6J mice and AML12 cells, and the gene expression was verified by RT‐PCR. In summary, SLC16A1, ENO3 and LEPR are satisfactory indicators to evaluate the pathological results of NAFLD cluster and NAFLD patients. Nevertheless, the limitations of the current study should be acknowledged. More detailed clinical characteristics are needed to validate the performance of predictive models. A greater number of NAFLD samples is required to elucidate the accuracy of the clustering associated with cuproptosis. Finally, the potential correlation between CRG and immune response requires further investigation.

## 5 CONCLUSION

This study explored the correlation between CRGs and infiltrating immune cells and demonstrated the heterogeneity of infiltrating immune cells among NAFLD patients with different cuproptosis gene subgroups. Three characteristic genes (SLC16A1, ENO3 and LEPR) related to cuproptosis were identified. The present study confirmed the role of cuproptosis in NAFLD, further elucidated the underlying molecular mechanisms leading to NAFLD heterogeneity and provided new ideas for the development of new targets for immunotherapy in NAFLD patients.

## AUTHOR CONTRIBUTIONS

**Changxu Liu:** Visualization (equal); writing – original draft (equal); writing – review and editing (equal). **Zhihao Fang:** Visualization (equal); writing – original draft (equal). **Kai Yang:** Software (equal); validation (equal). **Yanchao Ji:** Data curation (equal); formal analysis (equal). **Xiaoxiao Yu:** Data curation (equal); resources (equal). **ZiHao Guo:** Investigation (equal); resources (equal). **Zhichao Dong:** Formal analysis (equal); investigation (equal). **Tong Zhu:** Investigation (equal); methodology (equal). **Chang Liu:** Funding acquisition (equal); methodology (equal); supervision (equal).

## CONFLICT OF INTEREST STATEMENT

The authors declare that they have no competing interests.

## Supporting information


Figure S1

Figure S2

## Data Availability

The datasets presented in this study can be found in online repositories. The names of the repositories and accession numbers can be found below: http://www.ncbi.nlm.nih.gov/geo/.
