# Supplementary material for: Identification and validation of cuproptosis‐related molecular clusters in non‐alcoholic fatty liver disease
Source: J Cell Mol Med. 2024 Jan 3;28(3):e18091. doi: 10.1111/jcmm.18091 (PMC10844703; doi:10.1111/jcmm.18091)
Supplement: Supplementary file 1 — Figure S1 [file JCMM-28-e18091-s002.zip › jcmm18091-sup-0002-FigureS1.docx]

**Figure S1**. Flowchart of this study.
