# Supplementary figures and images for: Identification and validation of cuproptosis‐related molecular clusters in non‐alcoholic fatty liver disease
Source: J Cell Mol Med. 2024 Jan 3;28(3):e18091. doi: 10.1111/jcmm.18091 (PMC10844703; doi:10.1111/jcmm.18091)

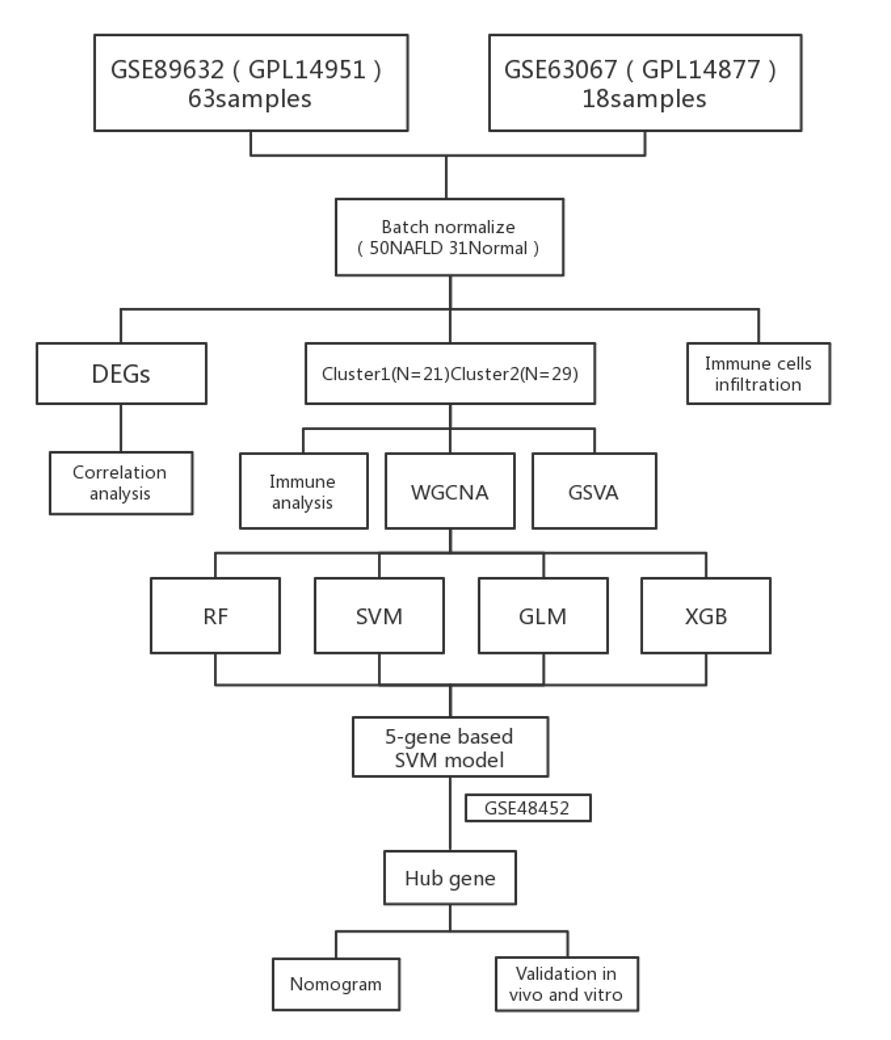

Supplement: Supplementary file 1 — Figure S1 [file JCMM-28-e18091-s002.zip › jcmm18091-sup-0001-FigureS1.jpg]

A

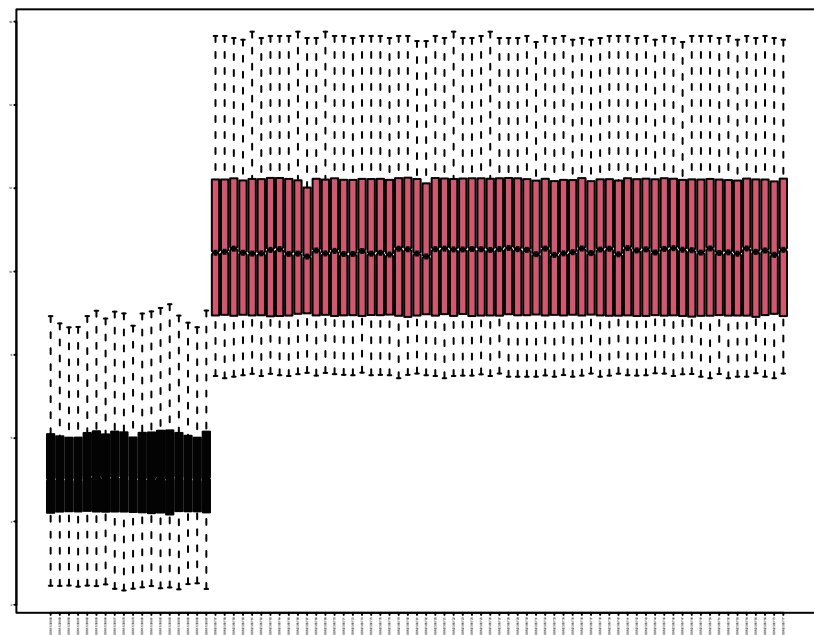

B

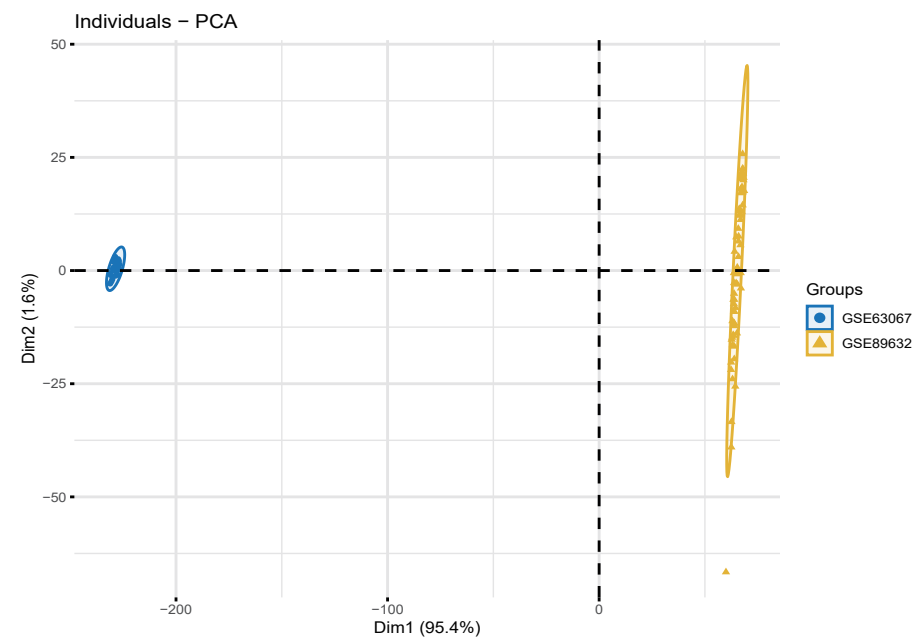

C

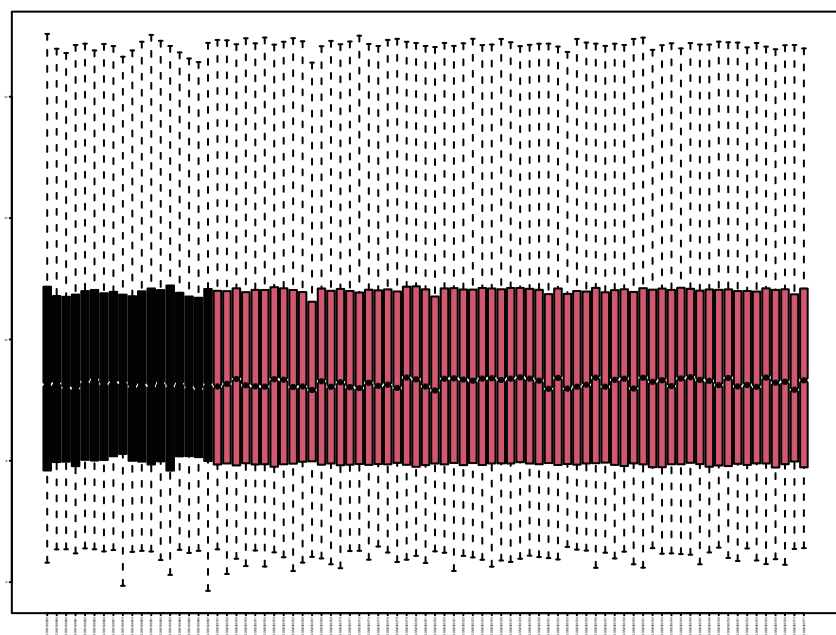

D

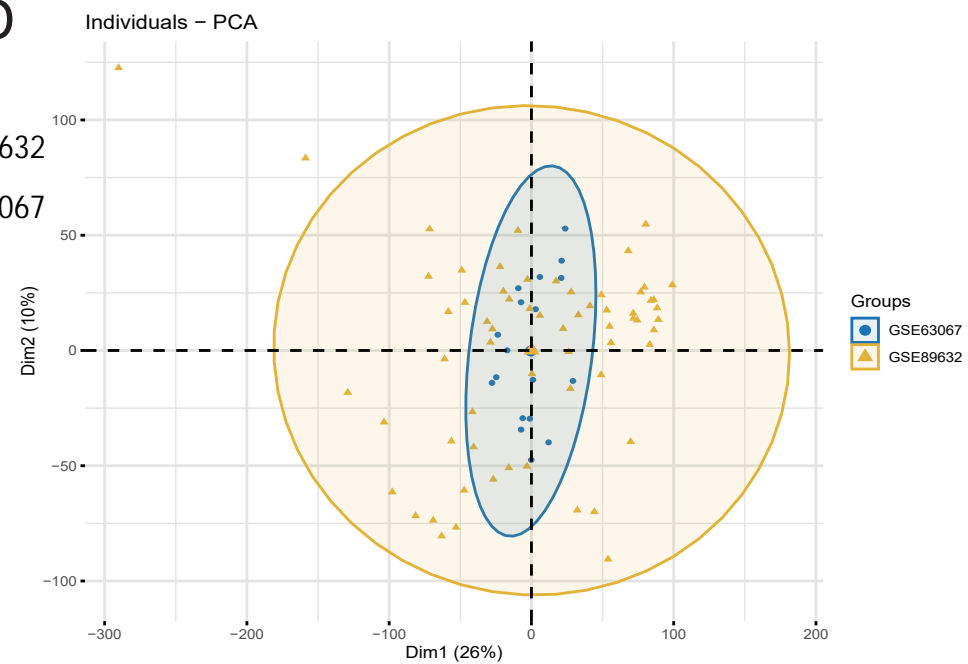

Supplement: Supplementary file 2 — Figure S2 [file JCMM-28-e18091-s001.zip › jcmm18091-sup-0002-FigureS2.pdf]
