# Supplementary material for: Identification and validation of cuproptosis‐related molecular clusters in non‐alcoholic fatty liver disease
Source: J Cell Mol Med. 2024 Jan 3;28(3):e18091. doi: 10.1111/jcmm.18091 (PMC10844703; doi:10.1111/jcmm.18091)
Supplement: Supplementary file 2 — Figure S2 [file JCMM-28-e18091-s001.zip › jcmm18091-sup-0001-FigureS2.docx]

**Figure S2.** Data preprocessing. (A, B) Box plot and principal component analyses were performed to remove batch correction of GSE63067 and GSE89632 before batch correction and (C, D) after batch correction.
